# Optical control of filamentation-induced damage to DNA by intense, ultrashort, near-infrared laser pulses

**DOI:** 10.1038/srep27515

**Published:** 2016-06-09

**Authors:** J. A. Dharmadhikari, A. K. Dharmadhikari, K. C. Kasuba, H. Bharambe, J. S. D’Souza, K. D. Rathod, D. Mathur

**Affiliations:** 1 Centre for Atomic and Molecular Physics, Manipal University, Manipal 576 104, India; 2 Tata Institute of Fundamental Research, 1 Homi Bhabha Road, Mumbai 400 005, India; 3 UM-DAE Centre for Excellence in Basic Sciences, Kalina Campus, Santacruz (East), Mumbai 400 098, India

## Abstract

We report on damage to DNA in an aqueous medium induced by ultrashort pulses of intense laser light of 800 nm wavelength. Focusing of such pulses, using lenses of various focal lengths, induces plasma formation within the aqueous medium. Such plasma can have a spatial extent that is far in excess of the Rayleigh range. In the case of water, the resulting ionization and dissociation gives rise to *in situ* generation of low-energy electrons and OH-radicals. Interactions of these with plasmid DNA produce nicks in the DNA backbone: single strand breaks (SSBs) are induced as are, at higher laser intensities, double strand breaks (DSBs). Under physiological conditions, the latter are not readily amenable to repair. Systematic quantification of SSBs and DSBs at different values of incident laser energy and under different external focusing conditions reveals that damage occurs in two distinct regimes. Numerical aperture is the experimental handle that delineates the two regimes, permitting simple optical control over the extent of DNA damage.

Due to ready availability of ultrashort pulsed laser sources, investigations of how such pulses propagate through transparent media have gained considerable contemporary research interest. The drivers for these investigations involve both the basic understanding of the underlying physics^1^,^2^,^3^ as well as the tantalizing prospects of a plethora of applications like remote sensing^4^,^5^ and remote control^6^ of processes that occur in the earth’s atmosphere, broadband spectroscopy^7^,^8^, modification of materials^9^,^10^,^11^,^12^, and bond-selective chemistry^13^. Interestingly, the potential for applications has, in recent years, begun to infringe upon the domain of the life sciences: experiments have been reported in which ultrashort, intense laser pulses have probed the possibility of non-invasively monitoring stress-related proteins in human saliva^14^; such pulses have also become of utility in medical applications like dental and eye surgery^15^. A break in a strand of DNA constitutes damage that can occur either naturally or via artificial means. Filamentation-induced damage has recently been demonstrated in biomolecules such as DNA kept under physiological conditions^16^,^17^,^18^. It has been suggested that detrimental dose distributions within tissue that are irradiated by gamma radiation - one of the major difficulties in radiotherapy - might be avoided by use of femtosecond laser induced filamentation^18^. This is due to ultrashort laser pulses, particularly in the infrared region, being spatially confined to volumes (~125 *μ*m^3^) that are very much smaller than what is possible to attain using contemporary clinical radiation sources. There is some evidence that 800 nm laser pulse induced filamentation can yield essentially the same radiation dosage in the radiolysis of water as that obtained using very energetic *γ*-radiation^19^.

Filamentation and supercontinuum generation are spatial and temporal manifestations, respectively, of how ultrafast pulses of intense light propagate through matter. Supercontinuum generation is a consequence of self-phase modulation (SPM)^20^,^21^ in tandem with a complex interplay of a gamut of processes, such as ionization-enhanced SPM^22^, four-wave parametric processes, self-steepening, group velocity dispersion and shock waves^23^,^24^,^25^,^26^,^27^,^28^,^29^. At incident power levels in excess of the critical power for self-focusing (typically ~4 MW for intense 800 nm light in water, and about three orders of magnitude larger in air) the optical Kerr effect causes the beam to self-focus. Upon reaching a small enough volume, the peak intensity of the self-focused beam can attain values that are high enough (~10^12^ W cm^−2^) to induce ionization of the medium, thereby creating electrons whose negative index contribution leads to defocusing of the beam. Along with diffraction, the dynamic balance that is set up leads to a series of focusing-defocusing cycles that enables the incident laser pulse to propagate to distances very much larger than the Rayleigh range, leaving behind a plasma channel with typical densities as large as ~10^18^ cm^−3^ being attained. Water molecules are ionized and dissociated within such a plasma channel, giving rise to low-energy electrons and OH-radicals^16^,^17^. These *in-situ* particles are utilized by us to probe electron- and radical-induced damage to DNA in an aqueous environment with a view to attaining an optical method to control the *extent* of damage that is induced, as described in the following.

In practice, filamentation, or formation of plasma channels, is achieved by externally focusing the incident laser beam. Competition between optical breakdown and filamentation in water was first investigated by Chin and coworkers^30^ in experiments that established the possibility of utilizing external focusing conditions to yield filamentation without breakdown, breakdown without filamentation, and filamentation with breakdown. Values of NA used in these studies spanned the range from 0.034 to 0.231. Theoretical simulations whose results are in accord with these experimental findings have subsequently been reported^31^. The effect of geometrical focusing on parameters like filament length within condensed media, such as a BaF_2_ crystal^32^ and plasma density in air^33^, has been studied. Very recently, a comprehensive numerical and experimental study was carried out on how filamentation in air can also be altered by the numerical aperture of the external optics^34^. Values of NA used in these studies spanned the range from 0.00085 to 0.011. Two distinct regimes were identified which depend on NA. For high values of NA, external (geometrical) focusing as well as plasma effects govern the filamentation dynamics. On the other hand, at low values of NA, it is the Kerr nonlinearity - that underpins the self-focusing-defocusing cycle referred to above - that dominates filamentation dynamics. The transition value of NA delineates linear and nonlinear focusing regimes, with different physical mechanisms dominating the dynamics in the two regimes. We explore here the possibility of utilizing external optics to affect the type of damage induced in DNA (SSBs or DSBs) and its extent. Our experiments are carried out in water and conducted at an order of magnitude higher values of NA than those used earlier. Values of NA used in our experiments spanned the range from 0.015 to 0.09. As has been shown earlier^16^,^17^, simple considerations of nonlinear absorption of incident laser light fail to properly account for the dynamics that drive plasma-mediated DNA damage. The results that we present offer clear indications that the extent and nature of DNA damage can be controlled optically simply by altering the numerical aperture of the external optics. We believe that our results provide a ready handle for optimizing this laser-based ionizing source for biological and biomedical applications.

## Results and Discussion

**Figure 1:**
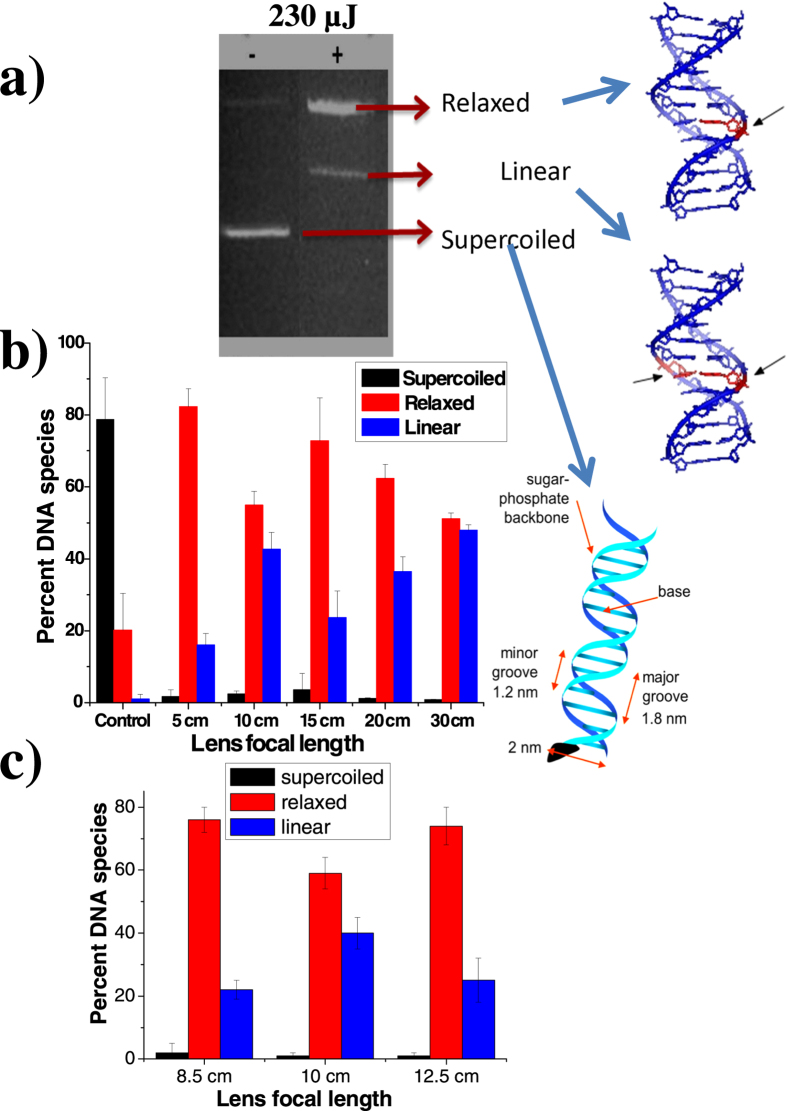
(**a**) Gel images obtained after pBR322 plasmid was irradiated with laser light using a 10 cm focal length lens. The negative and positive signs above the image panel indicate, respectively, no laser exposure and laser exposure for 180 seconds. Also shown are schematic depictions of single strand breaks (SSBs) and double strand breaks (DSBs) induced upon laser irradiation. Linear DNA results from DSBs. (**b**) Dependence of the percentage of DNA in supercoiled, relaxed, and linear states on the focal length of the external lens. The bars marked “Control” pertain to DNA prior to irradiation. (**c**) Dependence of the percentage of DNA in supercoiled, relaxed, and linear states for focal lengths of 8.5 cm to 12.5 cm. In each case irradiation was for 180 seconds using 820 nm laser light with the laser energy kept fixed (230 *μ*J) for different lenses.

We exposed plasmid DNA (pBR322) to plasma channels created in water (in which the plasmids were suspended). The extent of resulting damage was quantified using gel electrophoresis. As has already been reported by us^16^,^17^, formation of bubbles (including microbubbles) accompanies formation of the plasma channel over the range of irradiance values achieved in our experiments. In the present experiments, bubbles were clearly visible over the range of incident laser energies we used, for all NA values. However, at the lowest laser energies, bubbles were not always clearly visible: microbubbles were formed which had to be imaged on a CCD camera using a microscope objective. The time evolution of bubble diameter as a function of irradiation conditions follows complex dynamics^35^ and results pertaining to the present experimental conditions will be presented elsewhere. Under normal conditions, for a given preparation of plasmid DNA, around 80–99% of DNA are expected to be in their usual supercoiled state. A schematic depiction of such supercoiled geometry is shown in Fig. 1a. Between 1% to 20% of the population is usually found to possess a relaxed, open-circular geometry which results from single stand breaks (SSBs) that may be induced by a host of extraneous events (including handling of DNA in the course of preparation, interactions with cosmic rays, ultraviolet radiation, oxidizing agents, and such like). Our results (Fig. 1b) show that upon irradiation by 800 nm pulses (for up to 180 s) the resulting conformational changes are dramatic, with more than 80% becoming relaxed when the shortest focal length lens is used (generating the highest intensity); more than 50% become relaxed even at the lowest intensity that is obtained when we used the longest focal length lens (30 cm). Upon irradiation, less than 5% of DNA plasmids are seen to maintain their initial supercoiled structure. As many as 5% become linear. Our observations of the supercoiled → relaxed transformation are in agreement with earlier results obtained in near-IR experiments conducted at considerably lower intensity values^16^; tighter focusing and higher incident energy also permitted the occurrence of DSBs.

We observed that, in the case of 5 cm lenses, only SSBs are induced at incident energy values of 2 *μ*J. At higher energy values, DSBs also manifest themselves in the form of linear DNA, as seen in Fig. 1a obtained after 180 s exposure at an energy of 230 *μ*J. The linearization of DNA is a clear-cut signature of the occurrence of DSBs wherein two complementary strands of the DNA are simultaneously damaged. In the cellular context, this is the most lethal form of DNA damage, one that might lead to cell death or cancer if left unrepaired^36^. Within cells DSBs can occur due to many factors, such as oxidative damage by free radicals, ionizing radiation like X-rays^37^ and UV radiation^38^. DSBs generally constitute a small percent of the total damage^39^ but they are, of course, very pernicious. DSBs were, until recently, thought to be caused exclusively by high-energy radiation but recent work^16^,^17^ has shown that both SSBs and DSBs are induced within the laser-induced plasma channels formed in water. Thermal effects also induce SSBs, more so when longer laser wavelengths are employed^17^; however, they have no role to play in inducing DSBs.

Plasma formation upon propagation through water of intense (~100 TW cm^−2^) femtosecond laser pulses has been theoretically modeled^40^ by treating water as an amorphous semiconductor whose band gap is generally taken to be 6.5 eV^41^ although recent work has offered indications that the value is closer to 8 eV^42^. Ionization of water molecules occurs via both multiphoton absorption as well as tunneling; the ionized electrons are further accelerated by the optical field - by inverse Bremsstrahlung - before hydration sets in on relatively long time scales (in excess of a few picoseconds). In case of optical breakdown in water electron densities of 10^18^–10^20^ cm^−3^ have been deduced^40^. These low-energy (≤5 eV) electrons readily take part in dissociative attachment collisions with H_2_O: multiple transient negative ion states are formed within DNA which rapidly decay into damaged structures^19^,^43^,^44^,^45^. In contrast, high energy radiation induces such strand breakages mostly as a consequence of the sugar-phosphate backbone being ionized. Thus, femtosecond laser-induced breakdown may be regarded - in a loose sense - as resembling the effects of high energy ionizing radiation, such as *γ*-rays.

In our experiments on aqueous DNA, the key initiator of the damage-inducing dynamics is the strong optical field that is the precursor to excitation, ionization, and dissociation of H_2_O, yielding species like electronically excited H_2_O*, H_2_O^+^, OH, OH*, and low-energy ionized electrons^46^. Solvated electrons are long-lived enough to participate in the dynamics we describe here; their lifetime values are estimated to range from 300 ns^47^ to ~500 ps^48^. As discussed later, collisions between electrons and H_2_O can yield electronically excited H_2_O*. In turn, collisions between H_2_O* and H_2_O^+^ give rise to the formation of OH radicals, H_2_O* + H_2_O^+^ → OH + H_3_O^+^. Slow electrons, of specific energy, can also attach to H_2_O via a resonant process known as dissociative attachment, e + H_2_O → H_2_O^−^ → OH + H^−^. For instance, 7 eV electrons lead to formation of an H_2_O^−^ state that survives for a few hundred attoseconds^49^ before dissociating. It is the *slow* electrons and OH-radicals that are generated, *in situ*, in strong-field interactions with H_2_O that, in turn, induce transformation of DNA that we seek to explore.

**Figure 2:**
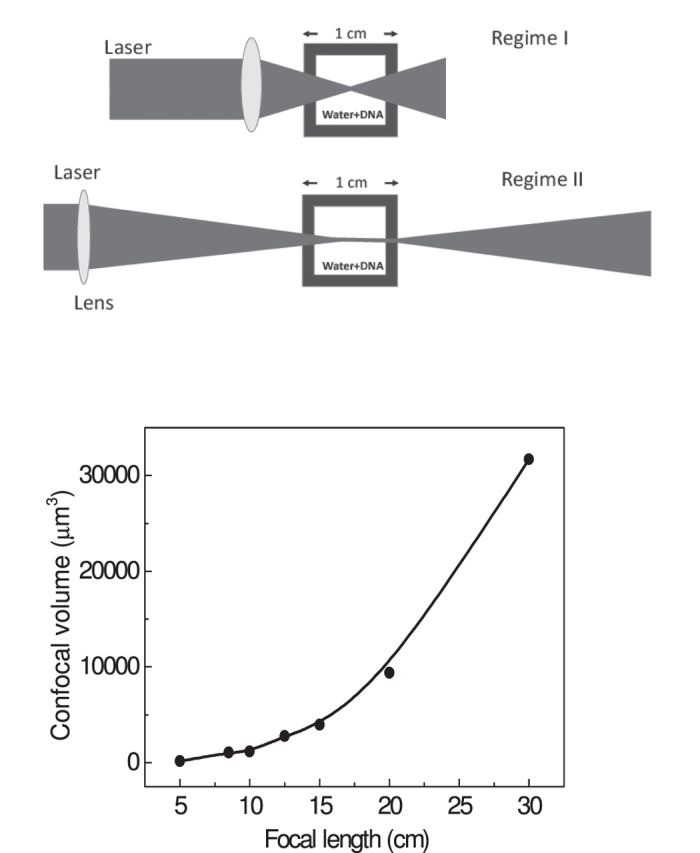
Upper panel: Cartoon depiction of Regime I and Regime II Lower panel: Variation of confocal volume on the focal length of the external lens.

Is it possible to exert experimental control over the extent of damage that is induced by ultrashort laser irradiation? We explore this possibility by quantifying the effect of external focusing of the laser beam that is incident on the water+DNA sample. Typical results are shown in Fig. 1b,c in which the percentage of supercoiled, relaxed and linear DNA is monitored as a function of the focal length of the lens used, keeping the incident laser energy at a fixed value (230 *μ*J). As the focal length is varied from 5 cm to 30 cm, the numerical aperture changes from 0.09 to 0.015. Perhaps more significantly from an experimental viewpoint, the confocal volume within which laser-DNA interactions take place changes from a compact 150 *μ*m^3^ for *f* = 5 cm to more than 32000 *μ*m^3^ for *f* = 30 cm. These numbers are computed without taking into account the fact that plasma formation, especially at high NA values, will make the effective confocal volume larger^30^, although the extent of such enhancement is difficult to quantify experimentally. The dependence of both parameters on focal length is shown in Fig. 2. The upper panel depicts, in cartoon form, two distinct regimes. At high NA values, where tight focusing is obtained using short focal length lenses, the interaction volume (confocal volume) is very small. On the other hand, for low NA values that are obtained when longer focal length lenses are used, the interaction (confocal) volume is larger: it takes the form of an extended plasma channel. For purposes of later discussion, we denote the high NA regime as Regime I and the low NA regime as Regime II. The observation that the percentage of relaxed and linear DNA does not change monotonically either with confocal volume or with incident energy indicates that an interplay of both factors determines the overall dynamics that cause strand breakages.

We note that the maximum energy to which the ionized electrons are accelerated is >5 eV at an intensity of 100 TW cm^−2^; it may be as high as a few hundred eV at 10 PW cm^−2^. Electron attachment is generally a resonant process but its overall cross section falls off very rapidly as electron energy increases. Thus, we anticipate that electrons play little or no role in inducing strand breakages in the high intensity regime that is accessed in our experiments. At incident energy of 2 *μ*J we observe SSBs while at 230 *μ*J we also observe DSBs in the case of the *f* = 5 cm lens. On increasing the focal length we observe an increase in the percentage of DSBs for the *f* = 10 cm lens but a reduction in DSB percentage for *f* = 15 cm. Further increase in focal length results in increase in DSB percentages.

**Figure 3:**
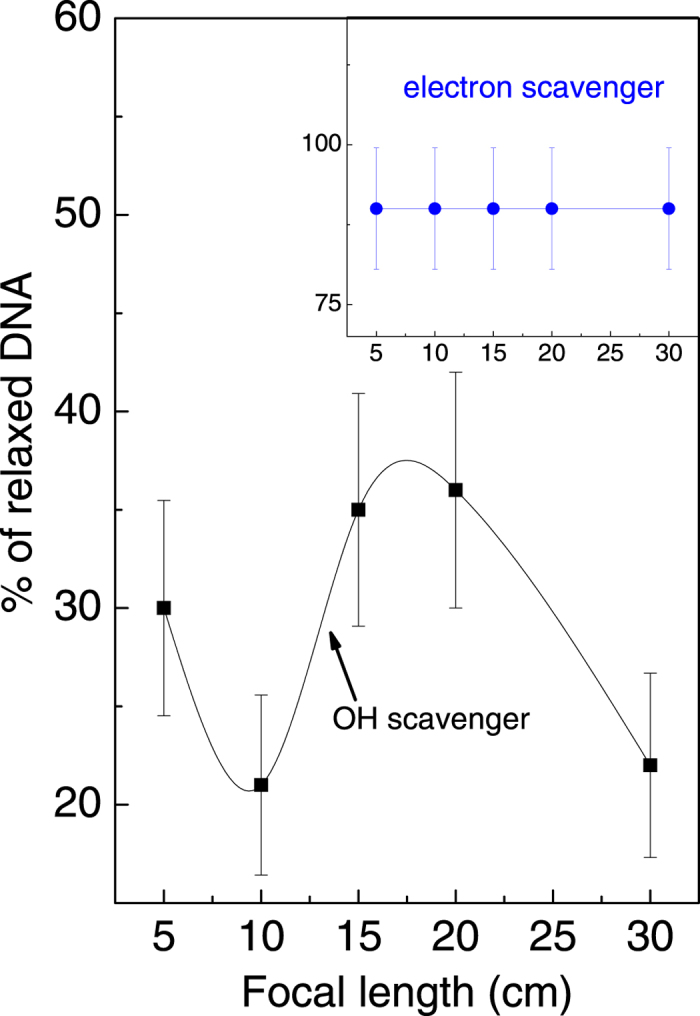
Variation in the percentage of relaxed DNA as a function of the focal length of the external lens in the presence of the OH-scavenger (sodium acetate). The inset shows the corresponding result obtained in the presence of the electron-scavenger (5-Bromouracil).

It is clear from our results (Fig. 1b,c) that there are two regimes that play a role in our experiments: the two regimes are delineated by NA. The two regimes are further exemplified in Fig. 3 where we discuss the biologically important result pertaining to linearization of initially supercoiled DNA by seeking an answer to the important question: Is it the electrons or the OH-radicals formed upon strong-field interactions with H_2_O that induce the conformational changes (supercoiled → relaxed, supercoiled → linear, and relaxed → linear) that we observe under different external focusing conditions? To probe this question we added electron- and OH-scavengers to the DNA+ water sample; sodium acetate is an OH-radical scavenger while 5-bromouracil is predominantly an electron scavenger. We investigated how DNA damage is affected in the presence of sodium acetate (over the concentration range 0–200 mM) and 5-bromouracil (over the concentration range 2–65 mM). On the basis of such concentration dependent measurements, we deduced that both electrons and OH radicals induce damage in DNA but that the latter is four times more pernicious than the former^16^. The relative invariance of percentages observed in relaxed DNA indicates clearly that electrons play little or no role in strand breakages that we observe in these experiments (inset of Fig. 3). As noted above, this is consistent with the electron energies under our experimental conditions being too high for attachment processes to occur with reasonable efficiency. In this context we note that higher-energy electrons (~7 eV) can, indeed, contribute to formation of H_2_O^−^ states^49^ but their ultrashort lifetime, of a few hundred attoseconds, preclude a significant role in inducing DNA damage. On the other hand, the results depicted in Fig. 3 show that the presence of the OH-scavenger strongly affects the percentages of relaxed species. Under our experimental conditions - high intensity irradiation by 800 nm light - it may be the OH-radicals that are overwhelmingly responsible for DNA strand breakages.

**Figure 4:**
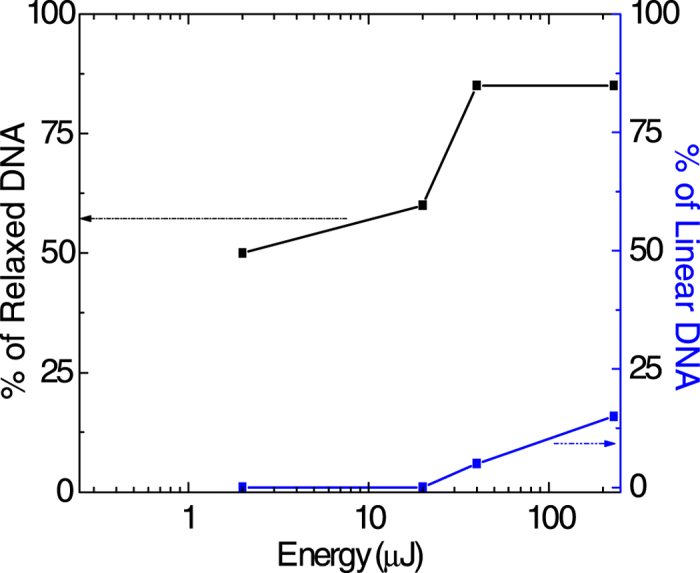
Variation in the percentage of relaxed and linear DNA as a function of incident laser energy.

Multiphoton excitation of DNA, which exhibits maximum linear absorption around 260 nm wavelength, might be expected to cause a variety of lesions, including DSBs^50^,^51^. However, our earlier experiments conducted at 1350 nm and 2200 nm wavelength^17^ have established that the extent of damage is not wavelength dependent and, consequently, multiphoton effects are unlikely to play a direct role in the strand breakage dynamics. Strand breakages are most likely induced by indirect effects that occur as the strong optical field interacts with H_2_O. Interactions of high energy x-rays and *γ*-rays with water give rise to OH formation which, in reactions with DNA, accounts for the majority of radiation damage to cellular systems^52^. Despite the reactions of OH radicals with the DNA (composed of a series of smaller molecules called nucleotides, with each nucleotide made up of nitrogenous base, sugar molecule called deoxyribose, and a phosphate group attached to the sugar molecule) have been investigated both experimentally and theoretically (see^53^,^54^, and references therein). However, the mechanism of OH-induced DNA damage are yet to be elucidated. Experimental evidence suggests that hydrogen abstraction mainly leads to damage in the form of SSBs which, as already noted, are amenable to repair. The occurence of DSBs, on the other hand, seems to require interactions involving electronically excited states of OH^17^ which are produced when H_2_O is electronically excited and then predissociates into OH*. For energies in excess of ~9 eV, direct dissociation of H_2_O* is adiabatically correlated to OH fragments in the excited *A*^2^∑^+^ state. In order to explore the efficacy of excited OH to induce DSBs, we made measurements at various values of incident laser energy. As is seen from the results shown in Fig. 4, measurable percentages of linear DNA are obtained only at laser energy in excess of 50 *μ*J. At lower energy levels it is likely that the energy gained by ionized electrons is insufficient to electronically excite H_2_O, precluding formation of excited OH*. The mechanism involved in OH*+DNA interactions leading to DSBs remains to be elucidated, mainly because of the currently intractable nature of the problem of understanding OH reactivity in an aqueous medium. The root of the problem arises due to the dynamics being dependent on the arrangement and conformations of *all* neighboring H_2_O molecules. It has been computationally demonstrated that by simply changing the water conformation the potential barrier for OH-induced hydrogen abstraction from a methane molecule alters by more than a factor of two^55^. Symptomatic of the difficulties of modeling is the computational demonstration in the case of guanine^53^ of the OH-induced hydrogen abstraction energy from the N_1_H or NH_2_ site increasing from its zero gas-phase value (indicating no barrier) to as much as 7–10 kcal/mol when guanine is solvated by only a dozen water molecules.

**Figure 5:**
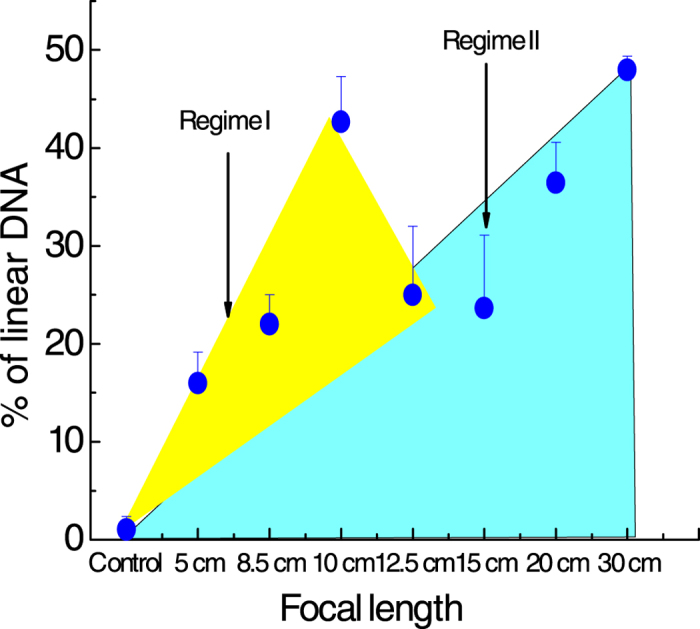
Variation in the percentage of linear DNA as a function of the focal length of the external lens. These measurements were made at an incident laser energy of 0.23 mJ. The value of numerical aperture (NA) for the 5 cm lens is 0.09; the corresponding value for the 30 cm lens is 0.015. As shown in Fig. 2, high NA values are denoted as Regime I while low NA values designate Regime II.

It is interesting to note in the context of our present work that OH-induced strand breakages are strongly dependent on external focusing conditions. This is clearly brought to the fore in results depicted in Fig. 5 where the percentage of linear DNA is plotted as a function of the focal length of the external focusing optics. These measurements were made at an incident energy of 230 *μ*J. We observe DSBs at NA values of around 0.09, obtained with a 5 cm lens. Increasing the focal length we observe an increase in the percentage of DSBs for a 10 cm lens (where the NA is 0.045). This case corresponds to the situation wherein both optical breakdown and filamentation are operative^30^. On reducing the NA, or increasing the focal length of the external lens, we observe a slight reduction in the DSB percentage which, upon further decrease in NA value (further increase in focal length) again produces an increase in DSB percentage. The functional dependence shown in Fig. 5 clearly allows demarcation of two distinct regimes: regimes I and II.

Experiments conducted using a 5 cm and 8.5 cm lenses show that geometric focusing plays a key role in restricting the region where low energy electrons and OH radicals are generated. For the 10 cm lens, we are operating close to the transition region where even though the effect of geometric focusing may be somewhat reduced, the Kerr focusing provides an extended region (Fig. 2) for generation of low energy electrons and OH radicals. At even higher focal lengths (12.5 cm and beyond) the Kerr focusing plays the dominant role; the external focusing appears to exert correspondingly less influence. In this regime electron energies can be large enough to induce formation of OH radicals in rotationally hot states. Although the intensities within the plasma channel may be clamped the accompanying spatial extension of the plasma channel in the Kerr focusing regime (Regime II) leads to a larger propensity for DSBs to occur. This is clearly reflected in our data pertaining to the occurrence of SSBs and DSBs as a function of the focal length of the external lens.

## Summary

In summary, we conducted experiments to probe damage to aqueous DNA upon interactions with low-energy electrons and OH-radicals produced in plasma channels formed in water. Our measurements have used DNA damage as a readout. Our results provide evidence for single and double strand breakages occurring in two distinct regimes: low NA and high NA. Our method relies on a novel use of strong-field interactions with water wherein electrons and free radicals are generated *in situ* upon multiphoton and tunneling ionization and dissociation of H_2_O. The low-energy electrons and OH radicals interact with DNA plasmids under physiological conditions, producing nicks. We quantify the damage caused by using electron and OH-scavengers. Our experiments offer indications that OH-radicals are mainly responsible for formation of DSBs, with a prominent role being played by electronically excited OH* radicals that are produced upon pre-dissociation of electronically excited H_2_O* states. Such electronically excited states of H_2_O are, of course, themselves formed from interactions involving electrons in the plasma channel that is induced in water upon intense laser irradiation. We have carried out systematic quantification of SSBs and DSBs at different values of incident laser intensity (keeping the focal length of the external lens constant) as well as under different external focusing conditions. We have demonstrated the feasibility of employing a simple optical method to vary the extent of damage in DNA. Our findings have implications beyond studies of damage to DNA *per se*. Our experimental technique of generating, *in situ*, slow electrons and radicals within aqueous media has important implications in different scenarios where the effects of non-ionizing radiation need to be probed under physiologically relevant conditions.

## Methods

Ultrashort pulses of 800 nm laser light are generated from an Ti:sapphire amplifier operating at 1 kHz repetition rate that has been described in several recent reports^17^,^46^. Using spectral shear interferometry the incident laser pulse duration was measured to be 40 fs. The incident beam had a *M*^2^ value of 1.3. and the beam diameter was 9 mm. Different lenses of focal lengths in the range 5 to 30 cm were used to carry out irradiation of our DNA sample for a period of 180 seconds.

Our DNA (pBR322), obtained from a commercial source (Merck-Millipore, India). The samples were dispensed into convenient volumes and stored at −20 C. The concentration of DNA was spectrophotometrically determined and we standardized the amount of DNA to yield maximum nicking, establishing a working range of 2–6 × 10^11^ molecules in 300 *μℓ* sample volume. We found that the lower end of this range yielded the best percentage of relaxed species following laser irradiation for 180 s. The concentration of our plasmid DNA was measured to be in the range 1.9–3.8 × 10^11^ cm^−3^, corresponding to concentrations of 0.9–1.8 *μ*g per 300 *μℓ*, out of which ~3 × 10^8^ plasmids were expected to be within the plasma channel (the confocal volume) - constituting 0.03% of plasmids. Related work^16^,^17^ carried out in our laboratory has established that strong thermal gradients are set up as our intense laser beam propagates through water +DNA, giving rise to convective flow. Thus, DNA molecules within the confocal volume are constantly replenished.

After irradiation, DNA fragments were separated using gel electrophoresis. Post-separation, the gel was stained with a DNA binding fluorescent dye, ethidium bromide, which enabled us to image and carry out quantification using a BIORAD Gel Documentation system in conjunction with standard gel-analysis software (ImageJ). We made use of commercially available DNA ladders containing linear fragments of known length to identify the DNA fragments.

## Additional Information

**How to cite this article**: Dharmadhikari, J. A. *et al.* Optical control of filamentation-induced damage to DNA by intense, ultrashort, near-infrared laser pulses. *Sci. Rep.* **6**, 27515; doi: 10.1038/srep27515 (2016).
